# White Matter Hyperintensities on Brain MRI are Related to Brain Atrophy and Accelerated Brain Age

**DOI:** 10.1002/alz70862_110308

**Published:** 2025-12-23

**Authors:** Somayeh Meysami, Cyrus A. Raji, Soojin Lee, Saurabh Garg, Nasrin Akbari, Rodrigo Solis Pompa, Ahmed Gouda, Thanh Duc Nguyen, Saqib Basar, Yosef Gavriel Chodakiewitz, David A. Merrill, Amar Patel, Daniel J. Durand, Sam Hashemi

**Affiliations:** ^1^ Pacific Brain Health Center, Pacific Neuroscience Institute and Foundation, Santa Monica, CA USA; ^2^ Saint John's Cancer Institute at Providence Saint John's Health Center, Santa Monica, CA USA; ^3^ Mallinckrodt Institute of Radiology, Washington University in St. Louis, St. Louis, MO USA; ^4^ Vigilance Health Imaging Network Inc., Vancouver, BC Canada; ^5^ Prenuvo, Palo Alto, CA USA

## Abstract

**Background:**

Brain age – an estimate of chronological age‐ derived from structural brain MR neuroimaging may reveal underlying factors driving brain aging. White matter hyperintensities (WMH) are areas of abnormally high signal on FLAIR that frequently reflect chronic small vessel ischemic changes and potentially increased aging.

**Method:**

Overall, 1,164 healthy participants from four sites (mean chronological age 55.17 ± 12.37 years, 52% women; 48% men; 39% non‐white) were scanned on 1.5T MR machines with a whole‐body protocol. For each participant, a 2D multi‐slice FLAIR image was obtained. A 2D convolutional neural network, trained on data from 120 individuals across three public datasets (MICCAI 2017, ISLES2015, and ISLES2022), was employed to automatically segment WMH from the FLAIR scans. Deep learning with FastSurfer on MPRAGE trained on 134 participants aged 27‐66 and segmented 96 brain regions. Brain age was computed using a regression‐based 3D Simple Fully Convolutional Network trained on in‐house T1‐weighted MRI scans collected from 5,500 healthy individuals (, aged 18 to 89 years). Brain age gap (BAG) was computed by subtracting chronological age from brain age. Partial correlation and regression models evaluated the relationship between WMH normalized to total brain volume (gray matter and white matter), brain age, brain volumes controlling for age, sex, and total intracranial volume. Chronological age was not adjusted for in the brain age models to avoid collinearity.

**Result:**

Mean brain age was similar to chronological age (mean brain age = 56.04 ± 12.65, mean BAG = 0.69). The median of WMH were 1.4 mL (0.75‐2.51 mL). Increased WMH were related to lower brain volumes in the i) hippocampus (rp= ‐0.13, *p* =  1.174e‐05) ii) cerebral white matter (rp= ‐0.08, *p* = .004) iii) thalamus (rp= ‐0.16, *p* = 1.634e‐07). Additionally, increased WMH was related to larger cerebral ventricle size (rp= 0.28, *p* =  6.604e‐21). The regression model showed that increased WMH was related to increased brain age (t= 5.92, rp= .42, *p* <.001) and increased brain age gap (t= 4.07, rp= .12, *p* <.001).

**Conclusion:**

Increased WMH are related to brain atrophy – in both Alzheimer’s and non‐Alzheimer’s affected regions – and are also related to increased brain age and accelerated brain aging.

